# Sex and age interact to determine clinicopathologic differences in Alzheimer’s disease

**DOI:** 10.1007/s00401-018-1908-x

**Published:** 2018-09-15

**Authors:** Amanda M. Liesinger, Neill R. Graff-Radford, Ranjan Duara, Rickey E. Carter, Fadi S. Hanna Al-Shaikh, Shunsuke Koga, Kelly M. Hinkle, Sarah K. DiLello, McKenna F. Johnson, Adel Aziz, Nilufer Ertekin-Taner, Owen A. Ross, Dennis W. Dickson, Melissa E. Murray

**Affiliations:** 1 Departments of Neuroscience, Mayo Clinic, Jacksonville, FL 32224 USA; 2 Departments of and Neurology, Mayo Clinic, Jacksonville, FL USA; 3 Biomedical Statistics and Informatics, Mayo Clinic, Jacksonville, FL USA; 4 Wien Center for Alzheimer’s Disease and Memory Disorders, Mount Sinai Medical Center, Miami Beach, FL USA

**Keywords:** Alzheimer’s disease, Neuropathology, Sex, Gender, Neurofibrillary tangle, Atypical, Plaques, Age, Young onset, Late onset, Autopsy, Postmortem

## Abstract

Women reportedly make up two-thirds of Alzheimer’s disease (AD) dementia sufferers. Many estimates regarding AD, however, are based on clinical series lacking autopsy confirmation. The Florida Autopsied Multi-Ethnic (FLAME) cohort was queried for AD cases with a total of 1625 identified ranging in age from 53 to 102 years at death. Standard neuropathologic procedures were employed and clinical information was retrospectively collected. Clinicopathologic and genetic data (*MAPT* and *APOE*) were stratified by sex. Within the neuropathologically diagnosed AD cohort, the overall number of women and men did not differ. Men were younger at onset of cognitive symptoms, had a shorter disease duration, and more often had atypical (non-amnestic) clinical presentations. The frequency of autopsy-confirmed AD among women and men stratified by age at death revealed an inverse U-shaped curve in men and a U-shaped curve in women, with both curves having inflections at approximately 70 years of age. Regional counts of neurofibrillary tangles differed in women and men, especially when examined by age intervals. Women had overall greater severity of neurofibrillary tangle counts compared to men, especially in the hippocampus. Men were more often classified as hippocampal sparing AD, whereas limbic predominant AD was more common in women. Men and women did not differ in frequency of *MAPT* haplotype or *APOE* genotype. Atypical clinical presentations, younger age at onset and shorter disease duration were more frequent in men, suggesting that the lower reported frequency of AD in men may be due to more frequent atypical clinical presentations not recognized as AD. Our data suggest that neuropathologically diagnosed AD cases have the same frequency of women and men, but their clinical presentations and ages at onset tend to differ.

**Electronic supplementary material:**

The online version of this article (10.1007/s00401-018-1908-x) contains supplementary material, which is available to authorized users.

## Introduction

The strongest risk factor for Alzheimer’s disease (AD) is increasing age [38]. The impact of sex differences on AD risk between women and men, however, has been variably interpreted [3, 20, 28, 31, 37]. Evidence from multi-site studies suggests that women make up nearly two-thirds of patients with AD dementia in North America [28]. The prevalence of AD dementia among women and men has also been increasing steadily in European ethnic groups, particularly for women after age 90 [31, 37].

Postmortem studies have revealed that women tend to have greater AD pathology [5, 13], but men more often present with atypical clinical syndromes (e.g., frontotemporal dementia, corticobasal syndrome) [14, 24]. We developed a mathematical algorithm based upon density and distribution of neurofibrillary tangle counts to classify three neuropathologic subtypes of AD: hippocampal sparing AD, typical AD, and limbic predominant AD [24]. Further investigation revealed that 63% of the hippocampal sparing AD were men, while 69% of limbic predominant AD were women. Moreover, the hippocampal sparing AD subtype had an average age at onset of cognitive symptoms at 63 years and shorter average disease duration of 8 years. In contrast, limbic predominant AD had an older average age at onset of 76 years old and longer disease duration of 10 years. We found that nearly 50% of hippocampal sparing AD had an atypical (non-amnestic) clinical syndrome, whereas more than 90% of limbic predominant AD had an AD dementia clinical presentation.

We hypothesized that atypical clinical presentations may lead to more frequent clinical misdiagnosis of AD in men, and thereby result in an apparent reduced frequency of AD in men. Thus, we proposed to investigate differences in the frequency of AD utilizing an autopsy-confirmed series from the Florida Autopsied Multi-Ethnic (FLAME) cohort. The objectives of this study were: (1) to examine differences between women and men, with respect to demographics, clinical course, genetics, and neuropathologic measures; and (2) to examine sex differences in neuroanatomic distributions of amyloid-β plaques and neurofibrillary tangles stratified by age.

## Materials and methods

### Study samples

The FLAME cohort is based on a collection 2809 autopsied brains from the State of Florida Brain Bank [4], which is housed at Mayo Clinic in Jacksonville, Florida. The State of Florida Brain Bank is a component of the Alzheimer’s Disease Initiative supported in part by the State of Florida Department of Elder Affairs (http://elderaffairs.state.fl.us/doea/alz.php). From the *n* = 2809 available brains accessioned prior to August 2015, *n* = 1625 were neuropathologically diagnosed AD cases without known AD mutations [suppl. Fig. 1 (Online Resource 1)]. Further details on the cases are provided in the “Results” section. The autopsy-confirmed AD cases from the FLAME cohort are derived from a consecutive series of patients referred via memory disorder clinics distributed throughout Florida, community-based education seminars for caregivers of dementia patients, and Alzheimer’s Association educational support groups. It is important to note that clinical diagnosis was not used to include or exclude neuropathologically diagnosed AD cases. All research was done on postmortem samples that are regarded by the Mayo Clinic Institutional Review Board as exempt from the requirements of research on human subjects. All brains were acquired with appropriate ethical approval, and the study was approved by the Mayo Clinic Institutional Review Board (IRB 16-003061).

### Clinicopathologic assessment

Antemortem clinical history was retrospectively abstracted from available medical records, which included a brain bank questionnaire, medical history, neurologic examinations, and/or neurology or medical follow-up notes. Demographic information was recorded on the following with availability noted in parentheses: self-reported ethnoracial status [1625/1625 (100%)], family history of cognitive impairment [1576/1625 (97%)], years of education [908/1625 (56%)], and age at death [1625/1625 (100%)]. Age at onset of cognitive symptoms was abstracted by specifically reviewing the clinical history for evidence of cognitive impairment. Other neurologic presentations (e.g., parkinsonism, spasticity, muscle weakness) were not documented for age at onset. Cognitive complaints could include, but were not limited to issues with memory (e.g., repetitive conversation, spatial disorientation), problems with naming, word finding difficulty, difficulty with math, visuospatial skills, manipulation of objects, and/or behavioral changes [34]. The date on which cognitive symptoms were first noted was recorded and subtracted from date of birth to obtain age at onset in years, which was available for 1148/1625 (71%). The date of symptom onset was subtracted from date of death to obtain disease duration of cognitive symptoms in years, which was available for 1148/1625 (71%). All available Mini-Mental Status Examination (MMSE) dates and accompanying test scores (0–30 points) were recorded. There were 723/1625 (44%) cases with at least one MMSE test date and 465/1625 (29%) with two or more MMSE test dates. All MMSE test dates were subtracted from date of death to obtain a test interval before death. The final MMSE test score was assessed for those individuals who were tested within 3 years of death. Rate of cognitive decline was calculated as the reduction of MMSE points per year, using three or more MMSE test dates relative to date of death. The rate was calculated as the slope of the linear regression line through the MMSE test intervals (dependent variable) and MMSE test scores (independent variable). Clinical diagnosis was classified as non-AD or atypical if the patient was given an antemortem diagnosis of corticobasal syndrome, primary progressive aphasia, posterior cortical atrophy, frontotemporal dementia, dementia with Lewy bodies, or any of several less common diagnoses (e.g., Creutzfeldt-Jakob disease, dementia of unknown etiology, normal pressure hydrocephalus, Parkinson’s disease, Parkinson’s disease dementia, progressive supranuclear palsy, and vascular dementia).

**Fig. 1:**
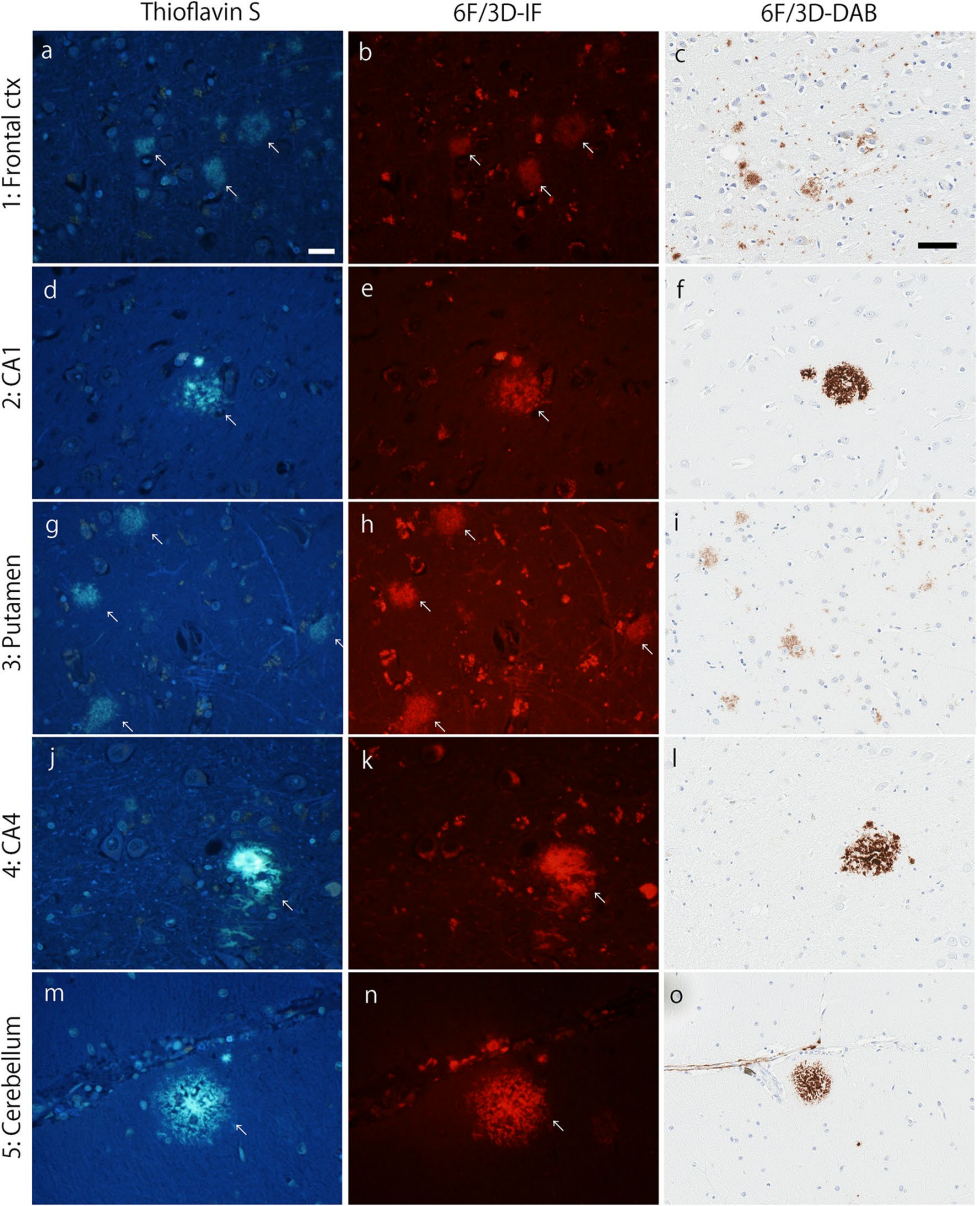
Thal amyloid phase. Representative images of thioflavin-S fluorescence (left column), immunofluorescent labeling of amyloid-β (6F/3D; middle column), and immunohistochemical 3,3′-diaminobenzidine (DAB) staining of 6F/3D (right column) in each Thal amyloid phase. Sections from the frontal cortex (phase 1; **a**–**c**), the pyramidal layer of the CA1 subsector of the hippocampus (phase 2; **d**–**f**), putamen (phase 3; **g**–**i**), CA4 subsector of the hippocampus (phase **j**–**l**), and the molecular layer of the cerebellum (phase **m**–**o**) are shown. Arrows indicate amyloid-β plaques that are labeled with both thioflavin-S and 6F/3D. Bars = 20 µm

**Fig. 2:**
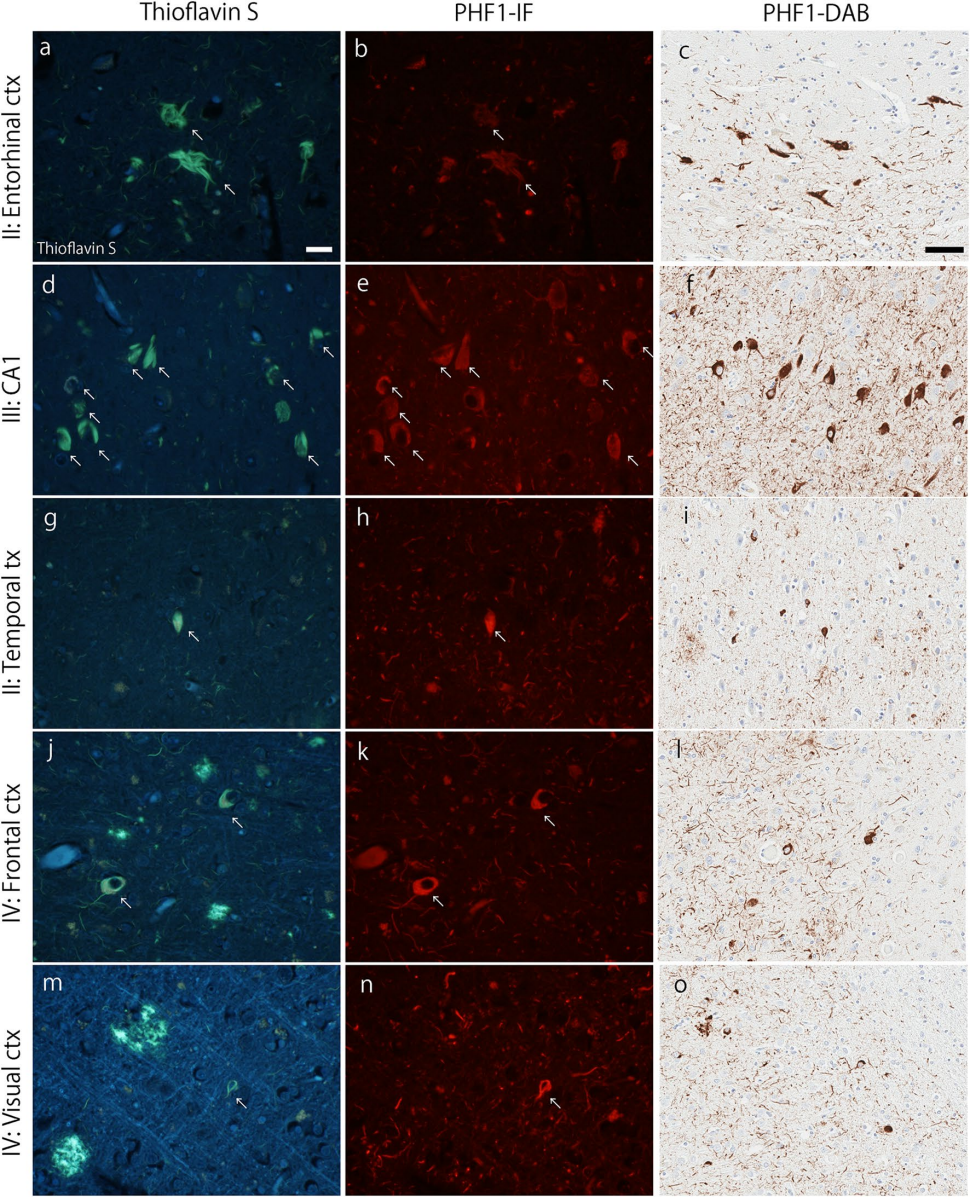
Braak tangle stage. Representative images of thioflavin-S fluorescence (left column), immunofluorescent labeling of tau (PHF-1; middle column), and immunohistochemical 3,3′-diaminobenzidine (DAB) staining of PHF-1 (right column) in each Braak tangle stage. Sections from the entorhinal cortex (stage II; **a**–**c**), the pyramidal layer of the CA1 subsector of the hippocampus (stage III; **d**–**f**), temporal cortex (stage IV; **g**–**i**), frontal cortex (stage V; **j**–**l**), and visual cortex (stage IV; **m**–**o**) are shown. Arrows indicate neurofibrillary tangle that are labeled with both thioflavin-S and PHF-1. Bars = 20 µm

Systematic and standardized neuropathologic examinations and procedures were performed as previously described [24]. Briefly, neuropathologic insults were quantified using thioflavin-S fluorescent microscopy to count neurofibrillary tangles and senile plaques in association cortices (frontal, temporal, and parietal), primary cortices (visual and motor), hippocampus (CA1 and subiculum), and amygdala; as well as the presence of these senile plaques in basal ganglia and cerebellum. Using an Olympus BH2 fluorescence microscope (Center Valley, PA, USA), slides were examined at low magnification to identify areas of greatest lesion density for plaques and tangles separately. All layers of cortex were reviewed with greatest density of plaques typically observed in the middle or upper layers and greatest density of tangles typically observed in layer III or layer V pyramidal layers. The pyramidal layer of the hippocampus is used to count plaques and tangles. Two or more microscopic fields are counted to find area of greatest density based upon visual assessment of lesion burden at low magnification. Senile plaques were counted using a 10 × objective (3 mm^2^ microscopic field) with 50 used as maximum, based on twice the density recommended to meet Khachaturian criteria for Alzheimer’s disease [17]. Neurofibrillary tangles were counted using a 40× objective (0.125 mm^2^ microscopic field) with no maximum. The data were analyzed and presented as counts per microscopic field. This information was then used to assign a Thal amyloid phase [36], Braak tangle stage [9], and AD subtype [24, 25]. Doublestaining of thioflavin-S and immunohistochemical markers was performed as previously described [18], but further details are provided in the suppl. Methods (Online Resource 2). Figures 1 and 2 provide representative images to illustrate overlap between thioflavin-S and immunofluorescent labeling with a primary antibody to amyloid-β or tau, respectively. Validation of our thioflavin-S neurofibrillary tangle findings was performed using PHF-1 (1:1000, mouse, anti-phospho-serine 396/404 tau, gift from Peter Davies). The presence of Lewy body disease was assessed using the antibody NACP (1:3000, rabbit, amino acids 98–115 with a cysteine residue at its C-terminus [7]). Lewy body disease subtypes were assessed based on neuroanatomic distribution of Lewy bodies [22]. TAR DNA binding protein 43 (TDP-43, rabbit, amino acids 220–227 in the 25-kDa C-terminal fragment [39]) pathology was assessed in the hippocampus for positivity [1]. Cerebrovascular disease was assessed based on methods used in the study by Jellinger and Attems (2003) [16]. Standard methods for genetic screening of *APOE* and *MAPT* were used, as previously described [24, 25].

To validate thioflavin-S findings, the hippocampus (CA1 and subiculum) and association cortices (frontal, temporal, and parietal) in 135 autopsy-confirmed AD cases were examined. PHF-1 slides were digitally scanned and analyzed using Aperio technology (Leica Biosystems, Buffalo Grove, IL), as previously described [24]. Briefly, the pyramidal layer of CA1 and subiculum were traced. The interface between the radiatum layer and molecular layer was used to define the superior border, while the alveus was used to define the inferior border. The association cortices were traced along the strait of the gyrus. A custom-designed color deconvolution macro was designed to recognize the strong intensity of neurofibrillary tangles to reduce quantification of the neuritic pathology. The quantitative data are summarized as a percent burden, which represents the percent of immunopositive labeling per mm^2^.

### Statistical analysis

SigmaPlot v12 was used to perform all statistical analyses. Significance was set at *p* value < 0.05. We assessed two-sample analyses using the Wilcoxon rank sum test, reporting the median and interquartile ranges. We assessed categorical variables using Chi square analyses, reporting number of cases (over appropriate denominator) and percent in category. Spearman Rank Order Correlation was performed to examine relationship between thioflavin-S and PHF-1 immunohistochemistry. Regression modeling of postmortem findings was performed to examine potential contribution of independent clinical parameters (age at onset, disease duration, education). Each neuropathologic variable was separately assessed as the independent variable using multiple linear regression for continuous variables and multiple logistic regression models for discrete variables.

### Role of the funding source

The sponsors of this study had no role in study design, collection of data, analysis of data, interpretation of data, or writing of the report. The corresponding author had full access to all the data in the study and had final responsibility for the decision to submit for publication.

## Results

### Stratification of men and women by clinical diagnostic group

Out of the 2809 autopsied FLAME study cases, regardless of clinical or neuropathologic diagnosis, there were *n* = 1435 (51%) men with an age range of 36–99 and *n* = 1374 (49%) women with an age range of 36–104. Within this cohort, *n* = 1625/2809 (58%) were neuropathologically diagnosed as AD with an age range of 53–102. Of the autopsy-confirmed AD, adequate clinical information was available on *n* = 1356/1625 cases (83%). These AD cases were stratified into three clinical diagnostic groups: (1) AD dementia, (2) AD dementia with a differential of an atypical clinical syndrome, or (3) atypical clinical syndrome [suppl. Fig. 1 (Online Resource 1), Table 1]. Among those who presented clinically with an AD dementia syndrome, 58% were women and 42% were men (*p* < 0.001); whereas, among those who presented with an atypical clinical syndrome, 38% were women and 62% were men (*p* < 0.001). In addition, cases presenting with an atypical syndrome were younger at age at onset (70 years) compared to those presenting with AD dementia (73 years) (*p* < 0.001). The cases presenting with atypical clinical syndromes also had a shorter disease duration (7.8 years) compared to those presenting with AD dementia (9.5 years) (*p* < 0.001). Although cognitive decline did not differ, the final MMSE score was lower in cases presenting with atypical clinical syndromes (12 points) compared to those presenting with AD dementia (15 points) (*p* = 0.048). Thal amyloid phase differed (*p* = 0.033), but was less evident where differences diverged as the medians reached Phase five for each group (*p* = 0.033). Braak tangle stage was lower in cases presenting with atypical syndromes (Stage V) compared to those presenting with AD dementia (Stage VI) (*p* < 0.001).

**Table 1:** Demographics and clinicopathologic findings of neuropathologically diagnosed AD cases stratified by clinical diagnostic grouping

	Clinical diagnosis of neuropathologically confirmed AD			*p* value
	AD dementia syndrome	AD dementia with A typical clinical syndrome	A typical clinical syndrome	
Demographic characteristics				
Sample size	993/1356 (73%)	166/1356 (12%)	197/1356 (15%)	
Women	577/993 (58%)	77/166 (46%)	76/197 (38%)	< 0.001^χ^
Men	416/993 (42%)	89/166 (54%)	121/197 (62%)	< 0.001^χ^
Education, years	14 (12,16)	14 (12,16)	16 (12,17)	< 0.001
Clinical findings				
Age onset, years	73 (67,79)	69 (62,75)	70 (62,76)	< 0.001
Disease duration, years	9.5 (6.6,13)	8.7 (7.0,11)	7.8 (5.4,10)	< 0.001
Cognitive decline, points/year	− 1.4 (− 3.4,− 0.16)	− 1.6 (− 4.5,− 0.22)	− 2.8 (− 5.5,− 0.21)	0.311
MMSE final score, points	15 (7,19)	11 (4,16)	12 (6,19)	0.048
Neuropathologic findings				
Thal amyloid phase	5 (5,5)	5 (5,5)	5 (5,5)	0.033
Braak tangle stage	VI (V,VI)	VI (V,VI)	V (IV–V,VI)	< 0.001

Unless noted, data are presented as median (interquartile range) and assessed in the subset of autopsy-confirmed AD cases from the FLAME cohort regardless of ethnoracial status. Significance tested using Rank Sum Test or ^χ^Chi square test where indicated

*AD* Alzheimer’s disease, *MMSE* Mini Mental State Examination

### Demographics, clinical findings, and genetic data in neuropathologically-diagnosed Alzheimer’s disease cases

A summary of the demographic, clinical, and genetic findings of autopsy-confirmed AD is found in (Table 2). Within the neuropathologically diagnosed AD cohort, the number of women and men did not significantly differ, with only *n* = 125 (8%) more women than men identified. A Chi square test was performed with the observed 46% men and 54% women against an assumed evenly divided cohort (i.e., 50% men and 50% women) did not reveal a significant difference (*χ*^2^ = 0.180, *df* = 1, *p* = 0.671). The lack of difference in frequency between men and women in an autopsy-confirmed AD cohort was consistent when the cases were stratified by ethnoracial status, with only 1 (5%) more man identified in the *n* = 19 black Americans and only five (7.5%) more women identified in the *n* = 67 Hispanic Americans. Education was lower in women (13 years for women vs. 16 years for men), whereas age at death (83 vs. 80 years) and age at onset (73 vs. 70 years) was greater in women (all *p* < 0.001). Disease duration was longer in women (10 vs. 8 years), and a greater proportion of women presented clinically with an AD dementia syndrome (79 vs. 66%) (both *p* < 0.001). Differences were not observed between women and men in frequency of family history of cognitive impairment, cognitive decline measured on longitudinal MMSE, or the final MMSE score. Neither *MAPT* H1H1 haplotype nor *APOE* ε4 genotype frequencies differed by sex.

**Table 2:** Demographics, clinical findings, and genetic data in neuropathologically diagnosed Alzheimer’s disease cases from the FLAME study

	Men	Women	*p* value
Demographic characteristics			
Sample size	750/1625 (46%)	875/1625 (54%)	
Ethnoracial composition^a^			0.850^χ^
Black American	10/750 (1%)	9/875 (1%)	
Hispanic American	31/750 (4%)	36/875 (4%)	
White American	709/750 (94%)	830/875 (95%)	
Family history, % positive	236/735 (32%)	279/841 (33%)	0.692^χ^
Education	16 (12,16)	13 (12,16)	< 0.001
Age at death, years	80 (73,84)	83 (78,88)	< 0.001
Clinical findings			
Age onset, years	70 (64,76)	73 (66,79)	< 0.001
Disease duration, years	8 (6,11)	10 (7,13)	< 0.001
Cognitive decline, pts/year^b^	− 1 (− 4,− 0.2)	− 1 (− 4,− 0.2)	0.672
MMSE final score, points^c^	13 (6,19)	12 (6,19)	0.860
Clinical diagnosis, %			< 0.001^χ^
AD dementia syndrome	416/626 (66%)	577/730 (79%)	
AD with a typical clinical syndrome	89/626 (14%)	77/730 (11%)	
Atypical clinical syndrome	121/626 (20%)	76/730 (10%)	
Genetic findings			
*MAPT* H1H1, %positive	246/425 (58%)	312/508 (61%)	0.303^χ^
*APOE* ε4, %positive	335/550 (61%)	412/627 (66%)	0.100^χ^

*AD* Alzheimer’s disease, *MMSE* Mini Mental State Examination, *MAPT* Microtubule associated protein Tau, *APOE* Apolipoprotein E

^a^Ethnoracial group is self-reported

^b^Cognitive decline was measured in 130 women and 143 men who had three or more MMSE scores available for analysis

^c^Last MMSE score was tested within 3 years of death (time from test date to death did not differ) and available for 122 women and 181 men. Unless noted, data are presented as median (interquartile range) and assessed in the subset of autopsy-confirmed AD cases from the FLAME cohort regardless of ethnoracial status. Significance tested using Rank Sum Test or ^χ^Chi square test where indicated

### Postmortem findings in neuropathologically diagnosed Alzheimer’s disease cases

Given the significant differences between women and men with respect to age at onset, disease duration, and education, these variables were incorporated into regression models to account for the contribution of the observed clinical differences (Table 3). AD brains were smaller for women than men (980 vs. 1120 grams), and women had higher Braak tangle stage (VI vs. V–VI) (both adjusted *p* < 0.001). The Thal amyloid phase approached significance, with women having slightly more amyloid (adj. *p* = 0.055). Lewy body disease, especially diffuse Lewy body disease, was higher in men (overall 27% in men and 21% in women), but this did not survive adjustment. As expected [24], men were overrepresented in hippocampal sparing AD (14 vs. 7%) and women were more than twice as frequent in limbic predominant AD (18 vs. 9%) (adj. *p* = 0.052). A higher frequency of TDP-43 positivity was found in women (44 vs. 35%), but this difference did not survive after adjusting for clinical variables. The frequency of co-existing hippocampal sclerosis did not differ between women and men. Cerebrovascular disease was more common in women (29 vs. 22%), but did not survive after adjustment.

**Table 3:** Postmortem findings in neuropathologically diagnosed Alzheimer’s disease cases from the FLAME study

Multiple linear	Men	Women	Unadjusted model		Adjusted model	
			Coefficient (SE)	*p* value	Coefficient (SE)	*p* value
Brain weight (g)	1120 (1020,1210)	980 (900,1060)	141 (7.2)	< 0.001	131 (8.8)	< 0.001
Thal amyloid phase	5 (5,5)	5 (5,5)	− 0.065 (0.032)	0.039	− 0.085 (0.044)	0.055
Braak tangle stage	V–VI (V,VI)	VI (V,VI)	− 0.16 (0.033)	< 0.001	− 0.19 (0.044)	< 0.001
Lewy body disease			0.16 (0.056)	0.005	0.13 (0.080)	0.104
None	541/736 (73%)	686/866 (79%)				
Brainstem	7/736 (1%)	7/866 (1%)				
Transitional	70/736 (10%)	72/866 (8%)				
Diffuse	118/736 (16%)	101/866 (12%)				
AD subtype			− 0.16 (0.026)	< 0.001	0.069 (0.035)	0.052
Hippocampal sparing	94/653 (14%)	53/747 (7%)				
Typical	498/653 (76%)	562/747 (75%)				
Limbic	61/653 (9%)	132/747 (18%)				

Multiple logistic			Odds ratio (5%, 95%)	*p* value	Odds ratio (5%, 95%)	*p* value
TDP-43 positive	151/428 (35%)	226/511 (44%)	0.69 (0.53,0.90)	0.005	1.0 (0.70, 1.4)	0.968
Hippocampal sclerosis^a^	49/750 (6%)	73/875 (8%)	0.77 (0.53,1.12)	0.169	1.0 (0.63, 1.7)	0.855
Cerebrovascular disease	167/750 (22%)	253/875 (29%)	0.70 (0.56,0.88)	0.002	1.0 (1.0,1.0)	0.830

*g* Grams, *AD* Alzheimer’s disease, *TDP-43* Tar DNA binding protein-43

^a^All hippocampal sclerosis cases are TDP-43 positive. Unless noted, data are presented as median (interquartile range) and assessed in the subset of autopsy-confirmed AD cases from the FLAME cohort regardless of ethnoracial status. Multiple linear and multiple logistic regression modeling was used to adjust for the potential contribution of significant clinical parameters (age onset, disease duration, education), where neuropathologic variable was the dependent variable and sex (Female = 0, Male = 1) was input as an independent variable

**Fig. 3:**
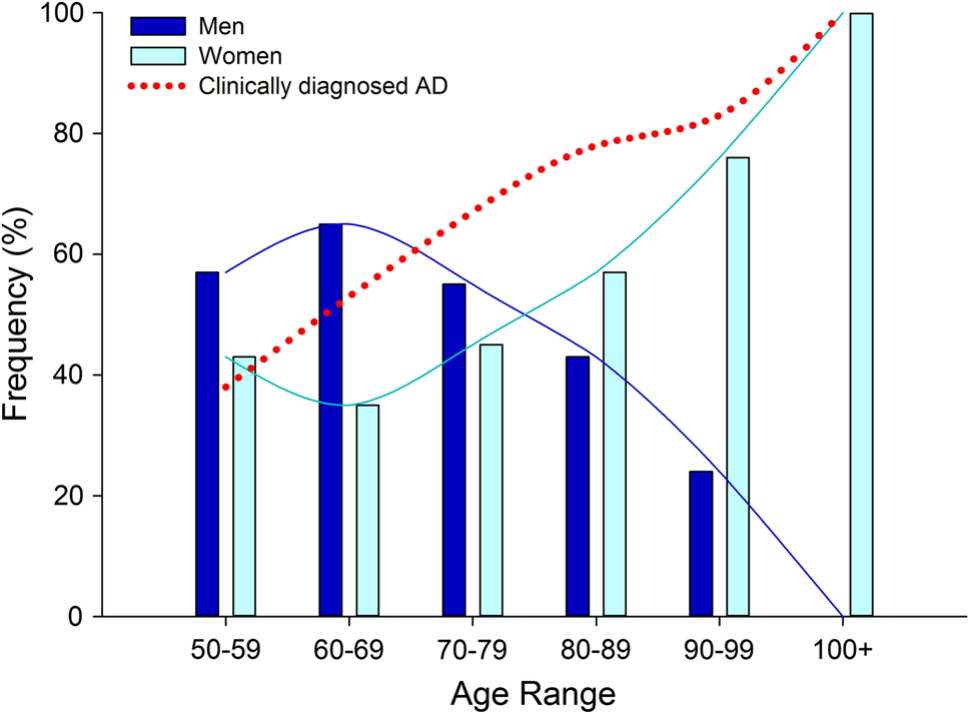
Disproportionate frequency of women and men neuropathologically diagnosed as Alzheimer’s disease across six decades. Frequency plots of age at death revealed an inverted U-shaped curve in men with autopsy-confirmed AD, demonstrating a higher frequency of death in their 7th decade. In comparison, age at death in women with autopsy-cofirmed AD was found to be overrepresented in later decades—particularly the 10th and 11th decades of life. The frequency of autopsied AD cases that presented clinically with an AD dementia without a non-AD or atypical clinical syndrome in the differential demonstrated a strong age-associated increase in the accuracy of the diagnosis

We examined the frequency of neuropathologic diagnosis of AD stratified by decade of age at death, ranging from the 6th (i.e., 50–59) to the 11th decade (i.e., 100–109) [suppl. Table 1 (Online Resource 3)]. Prior to the 9th decade of life, men with AD were overrepresented, with a peak frequency in the 7th decade of life. From the 9th decade of life onward, women with AD were overrepresented, with an observed exponential increase. No men with AD were observed in the centenarian cohort. Figure 3 illustrates frequency plots with an overlay of clinical presentation of AD dementia. Coinciding with an overrepresentation of women in the 9th decade, clinical presentation of AD dementia nearly doubled compared to that observed in the 6th decade of life.

### Neuropathologic distribution of amyloid-β plaque and neurofibrillary tangle by age and sex

**Fig. 4:**
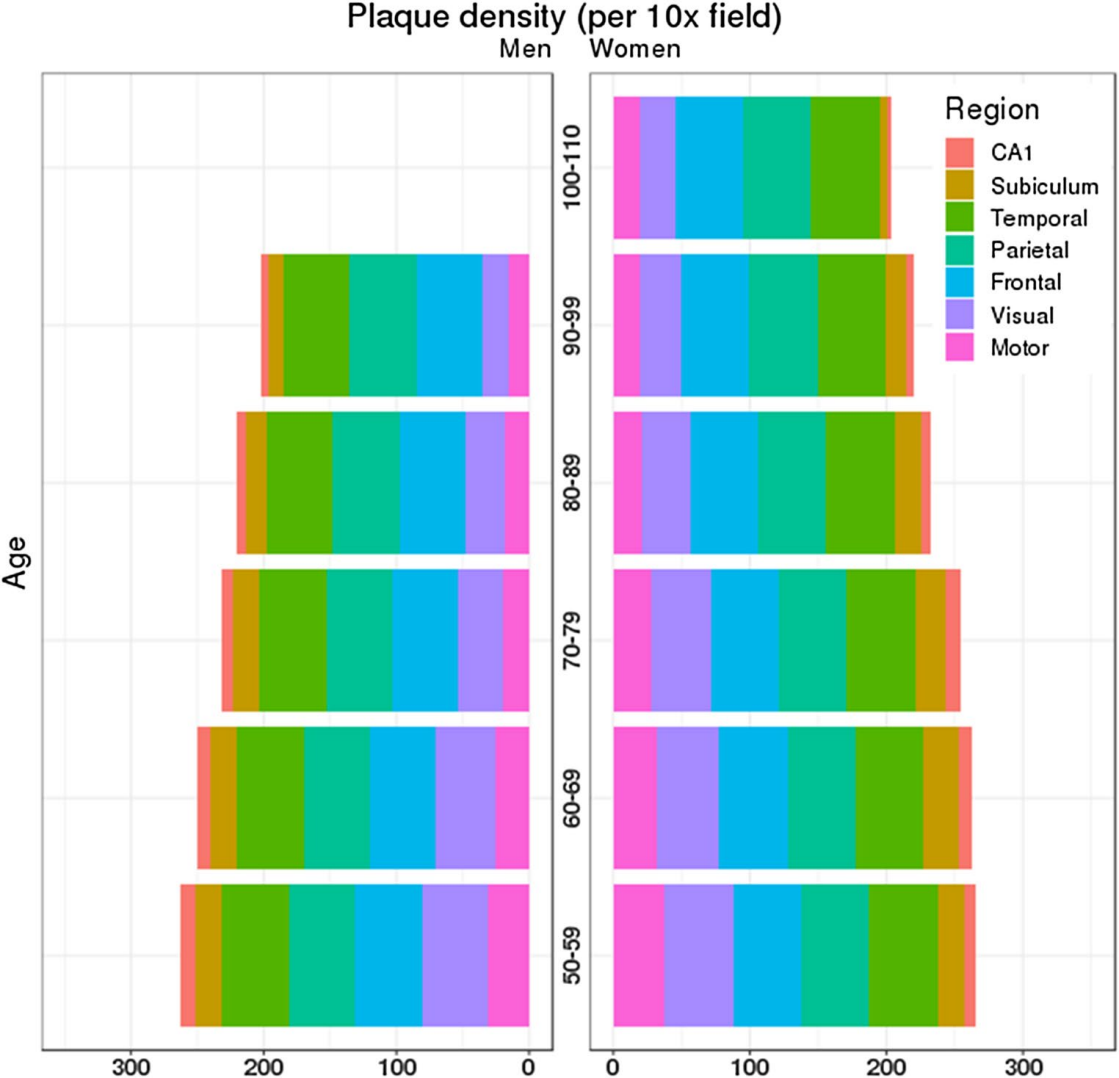
Cortical and hippocampal amyloid-β plaques quantitatively differ by age at death. Stacked bar charts graphically display the neuroanatomic distribution of amyloid-β plaque counts per 3 mm^2^. The colored cells represent the median value of amyloid-β plaque counts (x-axis) per given region (color coded), age at death (y-axis), and sex (right vs. left). The total width by age group is an additive sum of amyloid-β plaques, which demonstrates lessening pathology with advancing age. (Left) Men and (Right) women shared a similar pattern with a ceiling effect noted in the association cortices (frontal, temporal, and parietal). Both primary cortices (visual and motor) and hippocampal subregions (subiculum and CA1) were found to decrease as age increases

**Fig. 5:**
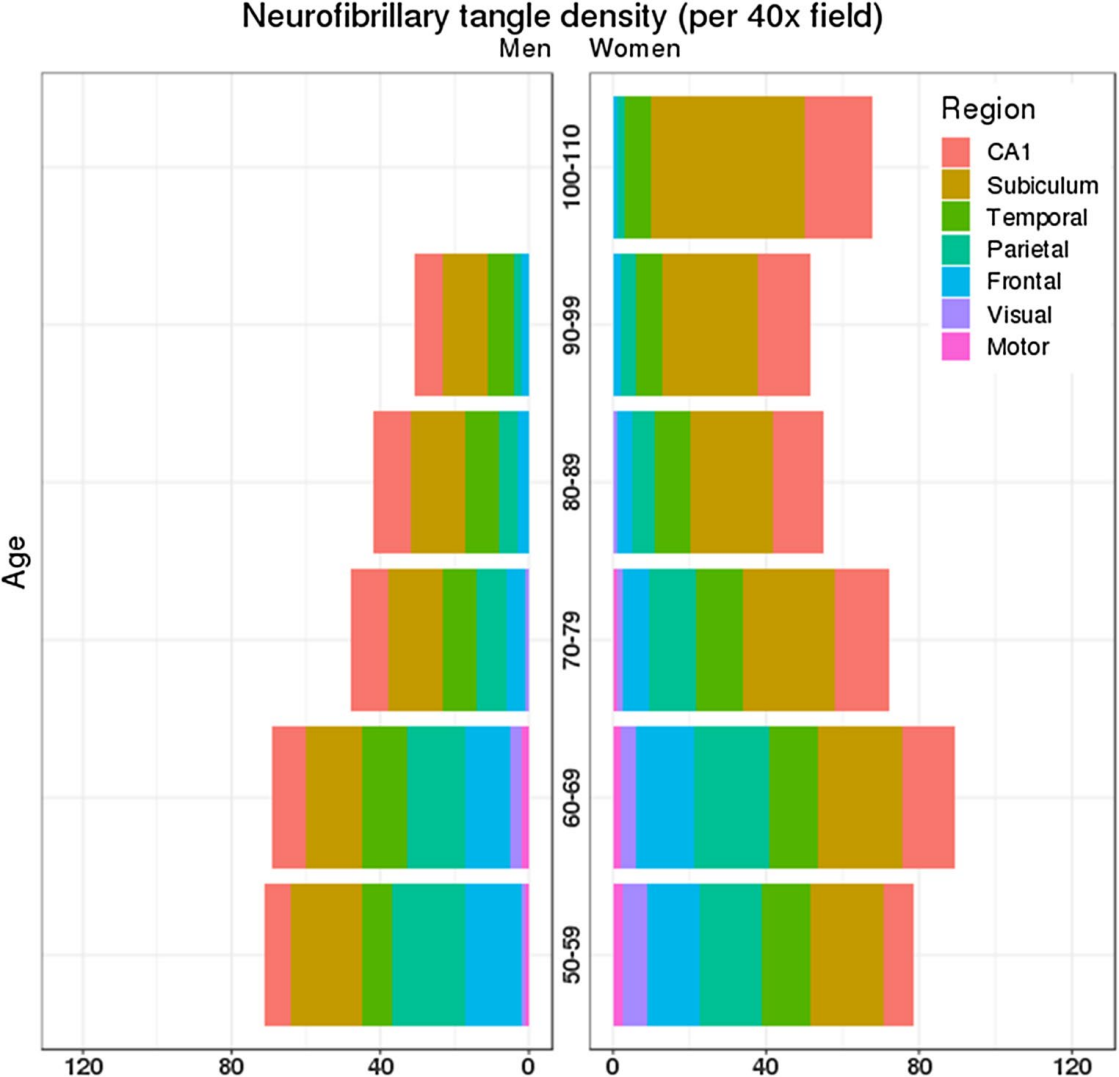
Cortical and hippocampal neurofibrillary tangles quantitatively differ by age at death and sex. Stacked bar charts graphically display the neuroanatomic distribution of neurofibrillary tangle counts per 0.125 mm^2^. The colored cells represent the median value of neurofibrillary tangle counts (x-axis) per given region (color coded), age at death (y-axis), and sex (right vs. left). The total width by age group is an additive sum of neurofibrillary tangles, which demonstrates lessening pathology with advancing age. (Left) Men and (Right) women differed in the age-associated involvement of the hippocampus, with women showing a more pronounced increase in their neurofibrillary tangle counts with advancing age. Both association and primary cortices were observed to have fewer tangles with each progressive decade in women and men. A shift between the 6th and 7th decades to the 8th decade onward was more pronounced in men, whereas the shift to fewer cortical tangles in women occurred more so in the 9th decade onward

Cortical and limbic amyloid-β plaque counts per 3 mm^2^ (10x magnification) (Fig. 4) and neurofibrillary tangle counts per 0.125 mm^2^ (40× magnification) (Fig. 5) in women and men were separately examined by terminal decade. Amyloid-β plaques were found to steadily decrease with advancing age across association cortices, primary cortices, and hippocampal regions. This pattern observed was similarly observed in men and women [suppl. Table 2 (Online Resource 3), Fig. 4]. Regional distribution of neurofibrillary tangle counts in the hippocampus and neocortex of men showed an overall pattern of a steady decrease in severity with increasing age [suppl. Table 3 (Online Resource 3), Fig. 5]. Neurofibrillary tangle counts in men remained higher in the neocortex during the 6th and 7th decades, with more than a twofold decrease observed in the 8th decade onwards. In contrast, neurofibrillary tangles increased steadily in hippocampal subregions of women with increasing age. Similar to men, neurofibrillary tangle severity decreased in the neocortex with increasing age, with a notable clustering in the 6th and 7th decade. A twofold decline was observed for women in the 9th decade onwards. The ratio between neurofibrillary tangles in women and neurofibrillary tangles in men within each brain region was stratified by age at death (Table 4). Women were more severely affected in the hippocampal-by-age matrix [9/10 (90%)]. This observation lessened in cortical regions as women were more severely affected in a little more than half of the association cortex-by-age matrix [9/15 (60%)] and less than half of the primary cortex-by-age matrix [4/10 (40%)]. In order to validate these findings, we examined age-associated sex differences in neuropathologic severity using immunohistochemistry in a subset of neuropathologically diagnosed Alzheimer’s disease cases. Thioflavin-S strongly correlated with PHF-1 immunopositive neurofibrillary tangle pathology in the CA1 (*R* = 0.774, *p* < 0.001), subiculum (*R* = 0.715, *p* < 0.001), superior temporal (*R* = 0.546, *p* < 0.001), inferior parietal (*R* = 0.785, *p* < 0.001), and middle frontal (*R* = 0.823, *p* < 0.001). A similar pattern of PHF-1 involvement was observed across neuroanatomic regions when examined by terminal decade in men and women [suppl. Table 4 (Online Resource 6**)**].

**Table 4:**
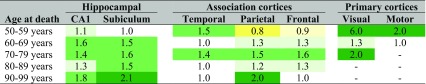
Ratio comparison of regionally distributed neurofibrillary tangles by age range between men and women in neuropathologically diagnosed Alzheimer’s disease

The median number of tangles in women was divided by the median number in men. Values < 1 (green scale) indicate greater severity in women, whereas values > 1 (yellow scale) indicate greater severity in men. A value = 1 indicates the same median value was identified in both men and women. Note: A dash (-) symbol indicates that the median tangle density was equal to 0 for both in men and women, with the exception of Visual tangles = 1 for women and = 0 for men

## Discussion

In the FLAME cohort, the overall frequency of autopsy-confirmed AD did not differ between women and men; however, men less often had an amnestic, multidomain clinical syndrome typical of AD dementia. As expected based upon societal gender-bias, women had lower educational levels. Women also had lower brain weights, older age at onset, longer disease duration, and older age at death. Severity of amyloid-β plaque pathology and neurofibrillary tangle pathology was greater in women compared to men. Interestingly, the frequency of autopsy-confirmed AD was highest among men in their 60 s, while women had an exponential increase from their 60 s into their late 90 s.

Our study investigating a large autopsy-confirmed AD cohort did not find an overrepresentation of women, but did find that men were less likely than women to present clinically with an AD dementia syndrome. A number of previous clinical studies, however, have found women are at a higher risk of AD than men [3, 20]. Others have shown that higher risk is more specific to older women [10, 15, 28, 31], supporting our observation of overrepresentation of women in their 80 s onward in an autopsy-confirmed AD cohort. Still others found no difference in incidence risk of AD in women, or once they controlled for age the risk was mitigated [19, 28, 29]. Collectively, these studies support our finding of overall similar frequency of AD in women and men.

Our study is one of the first to highlight over representation of autopsy-confirmed AD in men in the 7th decade (i.e., 60–69). Men were more likely to present clinically with a non-amnestic or atypical clinical syndrome (e.g., corticobasal syndrome, frontotemporal dementia) and to have a younger age at onset. Diagnostic accuracy of autopsy-confirmed AD was lower in men than women in this study, especially in the 6th and 7th decades, when men more commonly came to autopsy. Men were also found to have greater neocortical neurofibrillary tangle pathology, relative to hippocampal tangles compared to women. In contrast, women were overrepresented in later decades and had greater hippocampal neurofibrillary tangle pathology relative to neocortex. The above mentioned findings, which are also mirrored in tau PET biomarker studies [26], may be in concordance with the observation in epidemiological studies that mild cognitive impairment is more frequently diagnosed among men than in women [27]. One interpretation may be that women who have more severe memory impairment may abruptly transition from normal to a more severely impaired cognitive state, whereas men with atypical clinical syndromes may have a slower transition from a mild cognitive impairment state to dementia.

We and others have shown that the rate of cognitive decline does not differ between women and men, but that women are more likely to have overt dementia [5, 6]. Our data further demonstrate that neurofibrillary tangle counts in the hippocampus, across all ages, are more severe among women than men and that limbic predominant AD subtype is more common among women. Taken together with findings from neuroimaging studies demonstrating hippocampal atrophy occurs at a faster rate in women [2], our study supports the observation that women are more likely than men to present with a typical amnestic dementia. We hypothesize that women in later decades have a high frequency of correct diagnosis as AD dementia because AD neuropathology is more frequent in women and they have greater hippocampal involvement relative to the neocortex, leading to an amnestic syndrome.

In this study we extend our current knowledge by examining quantitative neurofibrillary tangle counts measures in association cortices, primary cortices, and hippocampal subsectors. Our findings suggest that there is an interaction between age and sex, resulting in selective neuroanatomic susceptibility to neurofibrillary pathology in different decades of life. Importantly, the neocortex may be especially vulnerable to neurofibrillary pathology in younger onset individuals, especially men, and to have a more rapid progression. Conversely, older aged individuals were found to have significantly reduced cortical pathology [8]. Women with autopsy-confirmed AD have been consistently found to be older at death [4, 5, 13, 32], but our study additionally suggests that men may be younger at onset of cognitive symptoms and have a shorter disease duration.

Our primary goal was to investigate the hypothesis that epidemiological studies showing greater prevalence of AD dementia in women may be biased by the sex- and age-influenced distribution of Alzheimer type pathology, which results in a disproportionate number of men with atypical clinical presentations. Our results support this hypothesis and point to differences in selective vulnerability to neurofibrillary tangle pathology as a plausible biological factor underlying differences in frequency of clinical diagnoses. Other biological factors that may have a bearing on our findings include the impact of sex hormones on neuronal vulnerability [30], as well as developmental differences in brain reserve [23, 33, 35]. One of the most important considerations when investigating sex differences in an aging cohort is differential survival of men and women, relating to their cardiovascular risk profile [10]. Thus, survival differences, resulting from differing cardiac risk profiles, could contribute to the observed lower frequency of AD in men in their later decades, as well as a greater frequency of men diagnosed with mild cognitive impairment.

This study has several limitations inherent to autopsy-based studies derived from specialty clinics or from self-referral. Although data were provided on clinical progression, the FLAME cohort is a cross-sectional and retrospective study. We did not observe overall sex differences in women and men, but there may be inherent bias among individuals who elected to participate in autopsy program [21]. Interestingly, we found that the overall FLAME cohort was comprised of 51% men and 49% women, suggesting brain donations from specialty clinics or from self-referral may not be sex-biased. Another potential limitation is that patients or the caregivers of patients with atypical clinical syndromes may be more likely to seek autopsy confirmation. Thus, our clinical findings may not be generalizable to population or epidemiologic-based cohorts. It should also be noted that our reported findings on cognitive decline were derived from a much smaller cohort with three or more available MMSE scores used to calculate rate of change. Thioflavin-S is a fluorescent dye that binds to β-pleated sheets and thus readily binds to both amyloid-β plaques and neurofibrillary tangles. Although immunohistochemical methods were used to validate thioflavin-S findings, it should be noted that differences in sensitivity may occur when assessing presence of pathology. An unbiased stereology approach was not employed to quantify neurofibrillary tangle and senile plaque data. To offset this limitation, neuropathologic lesions were quantified as counts per microscopic field across a large series of autopsy-confirmed AD cases. We did not observe sex-based frequency differences within black Americans or Hispanic Americans, but further examination of these underserved cohorts should be performed to identify whether our findings remain consistent in other diverse cohorts of autopsy-confirmed AD. Last, information on cause of death is rarely available, thus limiting further investigation into survival bias.

From a historical perspective, sex differences in research studies have not been adequately investigated and in some circumstances women have even been excluded from participating in many studies. This bias against inclusion of women ultimately prompted a federal mandate in the United States requiring inclusion of women in research studies [11]. Sex is often used as a covariate with balanced cohorts being investigated, but by statistically adjusting for sex, important biological clues underlying disease mechanisms may be neglected. Our data derived from autopsy-confirmed AD did not reveal a sex bias in terms of recruitment and consent for autopsy and they demonstrate that women and men have a similar frequency of AD. Sex-based differences in the age at onset, selective vulnerability of neocortical and limbic brain regions and the resulting clinical presentations may contribute to previous observations in clinical studies of AD being more common in women. These present observations should not take away from progress being made toward women’s health initiatives [12], but instead should be used to emphasize the importance of considering neuropathologic and biological differences in young-onset AD (i.e., < 65 years) compared to AD in the oldest-old (i.e., > 85 years). The bearing of sex and age, as well as differential rates of progression of memory impairment, may have important implications for therapeutic trials, which may benefit from stratification of women and men, and/or by age at onset when assessing efficacy.

## Electronic supplementary material

Below is the link to the electronic supplementary material.

Supplementary material 1 (DOCX 474 kb)

Supplementary material 2 (DOCX 17 kb)

Supplementary material 3 (DOCX 30 kb)
