# Supplementary material for: Sex and age interact to determine clinicopathologic differences in Alzheimer’s disease
Source: Acta Neuropathol. 2018 Sep 15;136(6):873–85. doi: 10.1007/s00401-018-1908-x (PMC6280837; doi:10.1007/s00401-018-1908-x)

**Supplementary Figure 1**. **Workflow of case material.** (Top) The overall FLorida Autopsied Multi-Ethnic (FLAME) cohort brain collection 2809 autopsied brains were investigated for neuropathologically diagnosed AD cases without known AD mutations. (Middle) The AD cases were part of a consecutive series that were evaluated in this study regardless of clinical diagnosis. (Left bifurcation) Toward the effort of understanding sex differences from the perspective of clinical syndrome, however, the autopsy-confirmed AD cohort was stratified by clinical syndrome. These AD cases were stratified into three clinical diagnostic groups: AD dementia, AD dementia with a differential of an atypical clinical syndrome, or atypical clinical syndrome. Table 1 summarizes demographics and clinicopathologic findings when the autopsy-confirmed AD cases were stratified by clinical syndrome. The frequency of autopsied AD cases that presented clinically with an AD dementia without a non-AD or atypical clinical syndrome in the differential was overlaid in Fig. 1. (Right bifurcation) The remaining text and figures, including Supplementary Tables presents data from the autopsy-confirmed AD cases regardless of clinical syndrome.


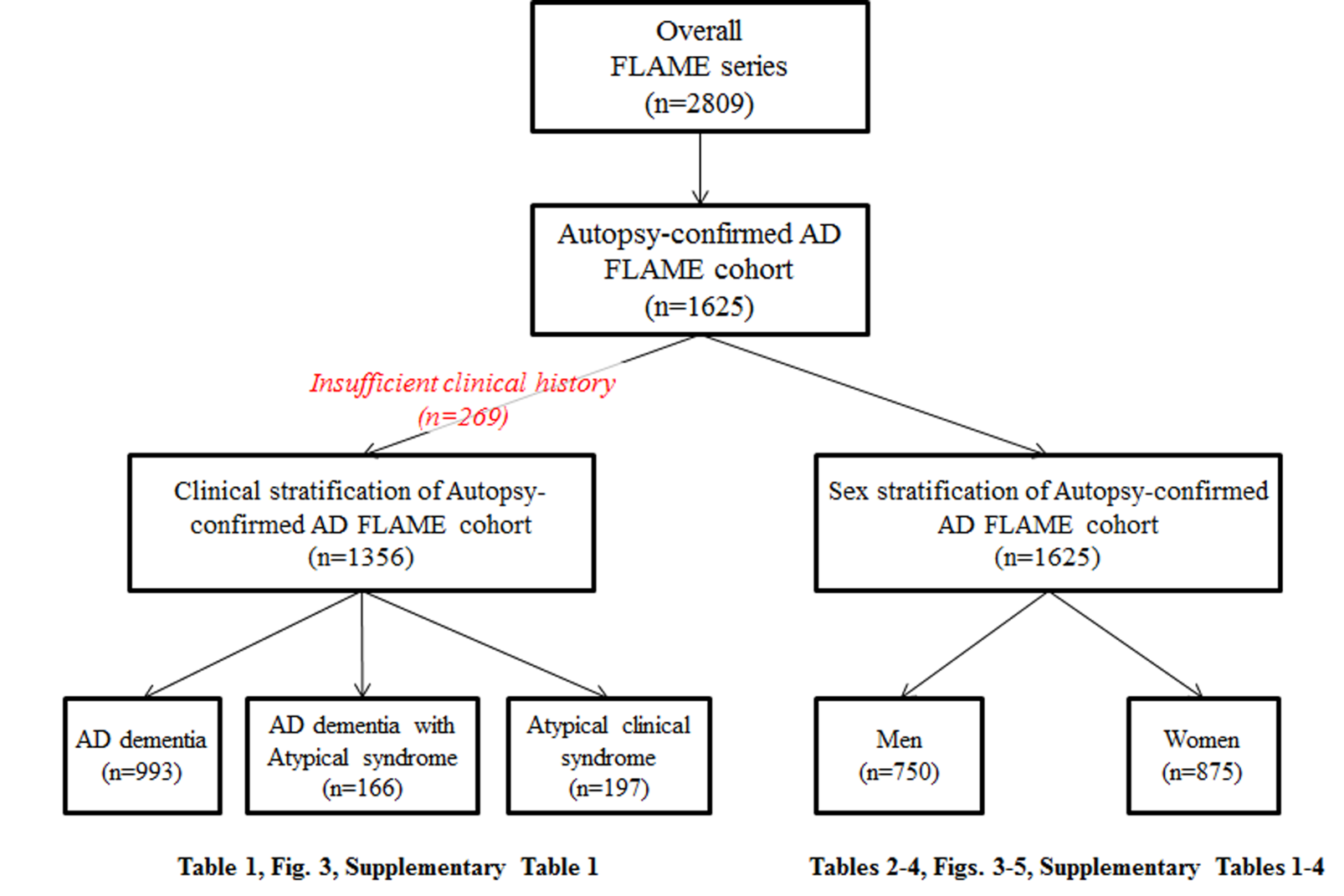

Supplement: Supplementary file 1 — Supplementary material 1 (DOCX 474 kb) [file 401_2018_1908_MOESM1_ESM.docx]
