# Supplementary material for: Sex and age interact to determine clinicopathologic differences in Alzheimer’s disease
Source: Acta Neuropathol. 2018 Sep 15;136(6):873–85. doi: 10.1007/s00401-018-1908-x (PMC6280837; doi:10.1007/s00401-018-1908-x)
Supplement: Supplementary file 2 — Supplementary material 2 (DOCX 17 kb) [file 401_2018_1908_MOESM2_ESM.docx]

**Online Resource 2**

R1c1

**Thioflavin S fluorescence and immunofluorescence doublestaining**

**Fig. 1** and **Fig. 2** provide representative images to illustrate overlap between thioflavin-S and immunofluorescent labeling with a primary antibody to amyloid-β or tau, respectively. Sequential labeling of thioflavin S and immunofluorescence with either 6F/3D (1:100, mouse, amino acids 8-17, Dako, Glostrup, Denmark) or PHF1 (1:1000, mouse, anti-phospho-serine 396/404 tau, gift from Peter Davies) was performed using a method described previously [*]. For Thal amyloid phase, sections from the frontal cortex (phase 1), the pyramidal layer of the CA1 subsector of the hippocampus (phase 2), putamen (phase 3), CA4 subsector of the hippocampus (phase 4), and the molecular layer of the cerebellum (phase 5) were used. For Braak tangle stage, sections from the entorhinal cortex (stage II), the pyramidal layer of the CA1 subsector of the hippocampus (stage III), temporal cortex (stage IV), frontal cortex (stage V), and primary visual cortex (stage VI) were used. Following deparaffinization, slides were incubated in thioflavin S solution for 7 minutes at room temperature. Subsequently, the slides were immersed in 70% ethanol using several dips for decolorization, and then immersed in deionized water for 1 minute. The slides were coverslipped with Vectashield mounting media (Vector Laboratories, Burlingame, CA). Thioflavin S fluorescence images were captured by BX50 fluorescent microscope (Olympus Co. Ltd., Tokyo, Japan) using U-MNU2 UV excitation fluorescence filter (Olympus Co. Ltd., excitation wavelength: 360-370 nm, emission wavelength: 420 nm). After capturing thioflavin-S images, the coverslip was removed. With the exception of a pretreatment step for 6F/3D of 95% formic acid for 30 minutes, the following steps apply to sections examined for representative figures of Thal amyloid phase (primary antibody = 6F/3D) and Braak tangle stage (PHF1). Next, the sections were steamed in distilled water for 30 minutes, blocked with Protein Block plus Serum Free (Dako) for 1 hour, and then incubated with primary antibody diluted with Antibody Diluent with Background-Reducing Components (Dako) overnight at 4°C. Sections were washed three times with 1xPBS (Sigma-Aldrich, St. Louis, MO) at room temperature, and then incubated with secondary antibody Fluor 568 (1:500, Thermo Fisher Scientific, Inc., Waltham, MA) and diluted with Antibody Diluent for 1.5 hours at room temperature in a dark chamber. Sections were washed three times with 1xPBS and mounted with Vectashield mounting media. Representative images were taken with the BX50 fluorescent microscope using M41002B green excitation fluorescence filter (Olympus Co. Ltd., excitation wavelength: 545 ± 30 nm, emission wavelength: 610 ± 35 nm). Adjacent sections from each case were processed for immunohistochemistry with anti-PHF1 (1:1000) or anti-6F3D (1:250) antibody using the Dako Autostainer.
