# Supplementary material for: Sex and age interact to determine clinicopathologic differences in Alzheimer’s disease
Source: Acta Neuropathol. 2018 Sep 15;136(6):873–85. doi: 10.1007/s00401-018-1908-x (PMC6280837; doi:10.1007/s00401-018-1908-x)
Supplement: Supplementary file 3 — Supplementary material 3 (DOCX 30 kb) [file 401_2018_1908_MOESM3_ESM.docx]

| **Online Resource 3:** Sex differences across decades in neuropathologically-diagnosed Alzheimer’s disease cases and their relevant clinical diagnosis of Alzheimer’s disease dementia. | | | | | |
| --- | --- | --- | --- | --- | --- |
|  | **Men** | **Women** | **p-value** | **AD clinically** | **p-value** |
| **Age at death*** | |  | <0.001 |  | <0.001 |
| 6^th^: 50-59 years (%) | 13/23 (57%) | 10/23 (43%) |  | 8/21 (38%) |  |
| 7^th^: 60-69 years (%) | 101/156 (65%) | 55/156 (35%) |  | 70/132 (53%) |  |
| 8^th^: 70-79 years (%) | 269/490 (55%) | 221/490 (45%) |  | 268/402 (67%) |  |
| 9^th^: 80-89 years (%) | 314/743 (43%) | 429/743 (57%) |  | 488/624 (78%) |  |
| 10^th^: 90-99 years (%) | 49/201 (24%) | 152/201 (76%) |  | 151/181 (83%) |  |
| 11^th^: 100-110 years (%) | 0/5 (0%) | 5/5 (100%) |  | 5/5 (100%) |  |
| Significance tested using chi-square test. *Age at death indicated by decade and years of age. | | | | | |

| **Online Resource 4:** Regional distribution of amyloid-β plaque pathology measured on thioflavin-S by age range in men with neuropathologically-diagnosed Alzheimer’s disease | | | | | | | |
| --- | --- | --- | --- | --- | --- | --- | --- |
|  | **Association cortices** | | | **Primary cortices** | | **Hippocampus** | |
|  | **Frontal** | **Parietal** | **Temporal** | **Visual** | **Motor** | **Subiculum** | **CA1** |
| **Age at death** |  |  |  |  |  |  |  |
| **Men** |  |  |  |  |  |  |  |
| 6^th^: 50-59 years (%) | 50 (50,50) | 50 (50,50) | 50 (50,50) | 50 (38,50) | 31 (26,47) | 20 (18,35) | 12 (7,22) |
| 7^th^: 60-69 years (%) | 50 (50,50) | 50 (50,50) | 50 (50,50) | 45 (35,50) | 25 (18,39) | 20 (12,31) | 10 (6,15) |
| 8^th^: 70-79 years (%) | 50 (50,50) | 50 (50,50) | 50 (50,50) | 33 (20,50) | 20 (14,30) | 20 (13,30) | 8 (5,12) |
| 9^th^: 80-89 years (%) | 50 (50,50) | 50 (50,50) | 50 (50,50) | 30 (18,40) | 18 (12,25) | 16 (10,25) | 7 (4,12) |
| 10^th^: 90-99 years (%) | 50 (50,50) | 50 (50,50) | 50 (50,50) | 20 (12,34) | 15 (10,25) | 12 (5,16) | 4 (2,8) |
| **P-value** | 0.597 | 0.542 | 0.123 | **<0.001** | **<0.001** | **<0.001** | **<0.001** |
| **Women** |  |  |  |  |  |  |  |
| 6^th^: 50-59 years (%) | 50 (50,50) | 50 (50,50) | 50 (50,50) | 50 (40,50) | 38 (30,46) | 20 (14,23) | 8 (6,16) |
| 7^th^: 60-69 years (%) | 50 (50,50) | 50 (50,50) | 50 (50,50) | 45 (34,50) | 32 (22,44) | 25 (16,32) | 10 (6,16) |
| 8^th^: 70-79 years (%) | 50 (50,50) | 50 (50,50) | 50 (50,50) | 43 (30,50) | 28 (18,40) | 23 (15,32) | 10 (6,15) |
| 9^th^: 80-89 years (%) | 50 (50,50) | 50 (50,50) | 50 (50,50) | 35 (22,48) | 21 (15,31) | 20 (12,30) | 7 (4,12) |
| 10^th^: 90-99 years (%) | 50 (50,50) | 50 (50,50) | 50 (50,50) | 30 (20,40) | 20 (14,29) | 15 (9,20) | 6 (4,10) |
| 11^th^: 100-110 years (%) | 50 (48,50) | 50 (50,50) | 50 (45,50) | 25 (16,32) | 20 (11,28) | 6 (3,14) | 3 (2,6) |
| **P-value** | 0.176 | 0.763 | **0.020** | **<0.001** | **<0.001** | **<0.001** | **<0.001** |
| Significance tested using chi-square test | | | | | | | |

| **Online Resource 5**: Regional distribution of neurofibrillary tangle pathology measured on thioflavin-S by age range in men with neuropathologically-diagnosed Alzheimer’s disease | | | | | | | |
| --- | --- | --- | --- | --- | --- | --- | --- |
|  | **Hippocampus** | | **Association cortices** | | | **Primary cortices** | |
|  | **CA1** | **Subiculum** | **Temporal** | **Parietal** | **Frontal** | **Visual** | **Motor** |
| **Age at death** |  |  |  |  |  |  |  |
| **Men** |  |  |  |  |  |  |  |
| 6^th^: 50-59 years (%) | 7 (3,14) | 19 (3,26) | 8 (5,12) | 20 (11,25) | 15 (12,19) | 1 (0,4) | 1 (0,2) |
| 7^th^: 60-69 years (%) | 9 (4,15) | 15 (8,25) | 12 (8,16) | 16 (12,24) | 12 (7,18) | 3 (1,5) | 2 (1,4) |
| 8^th^: 70-79 years (%) | 10 (5,16) | 15 (6,25) | 9 (4,15) | 8 (3,14) | 5 (2,11) | 1 (0,2) | 0 (0,1) |
| 9^th^: 80-89 years (%) | 10 (6,19) | 15 (7,27) | 9 (4,16) | 5 (2,10) | 3 (1,7) | 0 (0,2) | 0 (0,1) |
| 10^th^: 90-99 years (%) | 8 (4,17) | 12 (7,18) | 7 (3,12) | 2 (1,5) | 2 (1,4) | 0 (0,1) | 0 (0,1) |
| **P-value** | 0.100 | 0.313 | **<0.001** | **<0.001** | **<0.001** | **<0.001** | **<0.001** |
| **Women** |  |  |  |  |  |  |  |
| 6^th^: 50-59 years (%) | 8 (4,11) | 19 (8,28) | 12 (9,20) | 16 (14,20) | 14 (8,18) | 6 (5,10) | 2 (2,9) |
| 7^th^: 60-69 years (%) | 14 (7,20) | 22 (10,38) | 12 (7,22) | 20 (12,26) | 15 (8,25) | 4 (1,7) | 2 (1,4) |
| 8^th^: 70-79 years (%) | 14 (8,21) | 24 (12,35) | 13 (6,20) | 12 (5,18) | 8 (3,15) | 2 (0,5) | 1 (0,3) |
| 9^th^: 80-89 years (%) | 13 (8,22) | 22 (13,36) | 9 (4,16) | 6 (3,10) | 4 (2,8) | 1 (0,3) | 0 (0,1) |
| 10^th^: 90-99 years (%) | 14 (8,22) | 25 (14,42) | 7 (4,15) | 4 (2,8) | 2 (1,5) | 0 (0,2) | 0 (0,1) |
| 11^th^: 100-110 years (%) | 18 (12,22) | 40 (15,52) | 7 (2,11) | 2 (1,7) | 1 (0,4) | 0 (0,2) | 0 (0,0) |
| **P-value** | 0.159 | 0.380 | **<0.001** | **<0.001** | **<0.001** | **<0.001** | **<0.001** |
| Significance tested using chi-square test | | | | | | | |

| **Online Resource 6**: Regional distribution of neurofibrillary tangle pathology measured by PHF-1 by age range in men with neuropathologically-diagnosed Alzheimer’s disease | | | | | |
| --- | --- | --- | --- | --- | --- |
|  | **Hippocampus** | | **Association cortices** | | |
|  | **CA1** | **Subiculum** | **Temporal** | **Parietal** | **Frontal** |
| **Age at death** |  |  |  |  |  |
| **Men** |  |  |  |  |  |
| 6^th^: 50-59 years (%) | 0.45 (0.28,0.83) | 0.78 (0.42,1.1) | 0.45 (0.27,0.54) | - 1. (0.84,1.4) | 1.0 (0.90,1.2) |
| 7^th^: 60-69 years (%) | 0.89 (0.53,1.2) | 1.1 (0.80,2.3) | 0.59 (0.48,0.95) | 1.0 (0.86,1.3) | 0.84 (0.64,1.2) |
| 8^th^: 70-79 years (%) | 0.81 (0.51,1.2) | 1.2 (0.66,1.6) | 0.60 (0.33,0.85) | 0.72 (0.44,0.87) | 0.51 (0.17,0.73) |
| 9^th^: 80-89 years (%) | 1.1 (0.45,1.6) | 1.5 (0.81,2.4) | 0.52 (0.29,0.95) | 0.50 (0.24,1.0) | 0.28 (0.093,0.74) |
| 10^th^: 90-99 years (%) | 0.91 (0.89,0.96) | 1.4 (1.1,2.0) | 0.53 (0.40,1.3) | 0.42 (0.12,1.2) | 0.30 (0.022,0.43) |
| **P-value** | 0.487 | 0.418 | 0.727 | **0.012** | **0.001** |
| **Women** |  |  |  |  |  |
| 6^th^: 50-59 years (%) | 0.70 (0.18,1.2) | 0.59 (0.26,0.92) | 0.50 (0.28,0.72) | 1.8 (1.4,2.2) | 0.84 (0.52,1.2) |
| 7^th^: 60-69 years (%) | 1.1 (0.69,1.5) | 1.8 (1.4,2.7) | 1.0 (0.84,1.2) | 1.2 (0.73,1.5) | 1.1 (0.81,1.4) |
| 8^th^: 70-79 years (%) | 0.80 (0.48,1.2) | 1.6 (0.64,2.2) | 0.57 (0.26,0.68) | 0.57 (0.37,1.1) | 0.26 (0.17,1.0) |
| 9^th^: 80-89 years (%) | 1.4 (0.90,1.8) | 2.0 (1.4,2.5) | 0.48 (0.30,0.75) | 0.42 (0.21,0.59) | 0.21 (0.11,0.44) |
| 10^th^: 90-99 years (%) | 1.2 (0.76,1.6) | 1.6 (1.4,2.2) | 0.41 (0.27,0.64) | 0.20 (0.12,0.62) | 0.10 (0.036,0.40) |
| **P-value** | 0.168 | 0.187 | **0.085** | **<0.001** | **0.003** |
| Significance tested using chi-square test | | | | | |
